# Adsorption of Carvone and Limonene from Caraway essential oil onto Tunisian montmorillonite clay for pharmaceutical application

**DOI:** 10.1038/s41598-022-24268-5

**Published:** 2022-11-17

**Authors:** Chaima Agougui, Juan Antonio Cecilia, Houda Saad, Francisco Franco-Duro, Rym Essid, Mohamed Khabbouchi, Najoua Frini-Srasra

**Affiliations:** 1 Faculty of Sciences of Tunis (FST), Tunis El Manar University, Campus Universitaire Tunis El Manar, 2092 Tunis, Tunisia; 2 Laboratory of Composite Materials and Clay Minerals, National Center of Materials Research, Borj Cedria Technopole, Tunis, Tunisia; 3 Department of Inorganic Chemistry, Crystallography and Mineralogy, Universidad de Málaga, Campus de Teatinos, 29071 Malaga, Spain; 4 Laboratory of Bioactive Substances, Biotechnologie Center, Borj Cedria Technopole, Tunis, Tunisia

**Keywords:** Plant sciences, Environmental sciences, Chemistry

## Abstract

To explore a novel kind of green composite material having excellent antibacterial, antifungal ability and specific-targeting capability for pharmaceutical uses, a novel kind of bio-composite was prepared using sodium purified clay as carrier of Caraway essential oil (CEO). Gas chromatography-mass spectroscopy (GC–MS) analyses of CEO reveals that Carvone (68.30%) and Limonene (22.54%) are the two major components with a minimum inhibitory concentration (MIC) value equal to 125 mg/mL against *Staphylococcus (S) aureus* bacteria and *Candida albicans* fungi. Clay from Zaghouan was purified and characterized by X-ray Photoelectron Spectroscopy (XPS), X-ray diffraction (XRD), Fourier transformed infrared spectroscopy (FT-IR) and N_2_ adsorption–desorption (BET method). Results obtained by chromatograph equipped with a flame ionization detector (GC-FID) show that the concentration of 130 mg/mL of essential oil and 5 h of contact with the purified clay are the optimal conditions for the bio-hybrid formation. The pseudo-second-order model can describe the kinetic study of the adsorption of Carvone and Limonene on sodium montmorillonite, and the adsorption isotherms have been established to the Langmuir type. Limonene registers a maximum adsorption value equal to 3.05 mg/g of clay however Carvone register the higher amount of adsorption (19.98 mg/g) according to its polarity and the abundance of this compound in the crude CEO. X-ray diffraction, Fourier transformed infrared spectroscopy, elemental analyses (CHN) and X-ray fluorescence characterization valid the success adsorption of CEO in sodium montmorillonite surface. The purified clay/CEO hybrid (purified clay/CEO) combined the advantages of both the clay and the essential oil used in exerting the antibacterial and antifungal activity, and thus, the composite has a double antibacterial and antifungal activity compared to the separately uses of inactive clay and CEO, suggesting the great potential application in pharmaceutical treatments.

## Introduction

Essential oils (EOs) are volatile aromatic and hydrophobic oily concentrates obtained by *distillation methods, solvent extraction, enfleurage, and microwave-assisted extraction*. Moreover, the hydro distillation technique is one of the most effective techniques to extract the essential oil due to no consumption of organic solvents, high efficiency, and acceptable yield^1,2^ . In fact, studies have shown that the terpenoids and phenolic compounds are the essentially components, responsible to the biological properties of EOs^3^, which makes it widely used in pharmaceutical, cosmetic, agricultural and food industries^4–6^.

Focusing on the Caraway, the milling of its seeds leads to 7.5% of volatile oil, mainly Carvone and about 15% of fatty acids formed by palmitic, petroselinic, linoleic and oleic acids. Other products observed in the Caraway seeds are thymol, o-cymene, γ-terpinene, trimethylene dichloride, β-pinene, 2-(1-cyclohexenyl), cyclohexanone, β-phellandrene, 3-carene, α-thujene, and linalool^7^. Caraway essential oil exhibited interesting properties such as antibacterial, antifungal, antiparasitic and insecticidal^8^. The essential oil of caraway is mainly formed by Carvone and Limonene. Between them, Carvone possesses decongestant, diuretic, antiviral, and tonic biological properties^9^ while Limonene can be used to prevent bronchitis, carcinogenic processes, diabetes, gallstones, gastroesophageal reflux disease, heartburn and high cholesterol^10^.

Despite of all those benefits, the unplanned and inappropriate use of essential oils can lead to potential risks to human safety due to mutations, carcinogenic effects and genetic damage^11^. Like most other essential oils: Alkanet EO^12^; chamomile EO^13^ and Chili Pepper EO^14^, Caraway EO, can also cause skin rashes and itching in sensitive people when applied to the skin directly or when taken by mouth in food. Also, the higher volatility of essentials oils leads to a short duration of their activities. To overcome these problems, several researchers resort to direct incorporation of inedible /biodegradable emulsified films and coatings. But the disadvantage of this method is that the period of stability of the oils on it is not long and easy to volatilize and thus a loss of antimicrobial activity would take place. Another disadvantage is the use of toxic solvents like acetone^15^. The preparation of the hybrid based on essential oils and inorganic porous materials can be able to use essential oils safely without losing its benefits. The formation of this hybrid is generally realized by the process of adsorption. However, it is interesting to note that the conditions of adsorption have a significant effect on the formation of the hybrid, so that the investigation of this condition deserves to be investigating by theoretical and practical studies^16^.

The hybrids based on clays minerals and essential oils have received considerable attention due to their environmental compatibility, low cost, non-toxic, thermal stability, and recyclability^16,17^. Ghrab et al.^18^ used eucalyptus essential oil with modified beidellite by cationic surfactants in an insecticidal application, while Giannakas et al*.*^19^ employed oregano oil, thyme oil and basil essential oil with a purified and organo-modified montmorillonite for controlled release applications.

The montmorillonite clay is a sub-group of smectites, it is a 2:1 aluminum -magnesium phyllosilicate (or TOT type) composed of an octahedral sheet sandwiched between two silicon tetrahedral sheets. Isomorphic substitution in the octahedral sheets of the Al^3+^ by Mg^2+^ results in an overall negative charge, which is counterbalanced by interlayer cations such as Na^+^, K^+^, Ca^2+^, etc. The quantity of interlayer cation determines the cationic exchange capacity (CEC) of the sample^17^. Montmorillonite clay is characterized by their high surface area, ability to swell and cation exchange capacity as well as their layered structure, which makes them an ideal adsorbent abled to host cations or molecules in the interlayer spacing and/or on its surface^17^. In addition, it can be highlighted its use in gastrointestinal protectors, pharmaceutical formulations, dermatological protectors, cosmetics, oral and topical applications, anti-diarrheal, osmotic oral laxatives, excipients or controlled release of drugs^20^.

The aim of the present work is to synthesize an homoionic Na- montmorillonite/CEO hybrid (purified clay/CEO), characterized by better properties than pure essential oil, by using a green method without using a toxic organic solvent in order to make their usage easier in cosmetic and medical applications since the synthesized composite will be tested in the deactivation of a bacterium denoted as “*Staphylococcus (S.) aureus”,* an opportunistic pathogen, causes a variety of infections including impetigo, folliculitis, osteomyelitis, septic arthritis, septicaemia as well as endocarditis^21^, and a pathogenic fungus “*Candida albicans* agent responsible for superficial candidiasis^22^.

## Materials and methods

### Chemical materials

All reagents used in the present study were of analytical grade and used without further purification: (R)-Carvone (2-metyl-5-(pro-1-en-2yl) cyclohex-2-en-1-one) (96%) and (D)-Limonene (4-isopropenyl-1-methylcyclohexene) (97%) used as standards to calibrate the gas chromatography equipped with a flame ionization detector (GC-FID) were supplied by Sigma-Aldrich. Ethanol was supplied by (98%) from Sigma-Aldrich.

### Caraway essential oil (CEO) extraction

Caraway seeds were harvested from Korba in the north-east of Tunisia and provided by the Center of Biotechnology of Borj Cedria (Tunisia). The plant identification was confirmed by Prof. Abderrazzak Smaoui and a voucher specimen (Number 2525) was deposited in the herbarium at the Biotechnological Centre of Borj-Cedria.

The Caraway essential oil was obtained by hydro-distillation of the seeds during 3 h using a Clevenger-type apparatus. CEO was collected, centrifuged in order to eliminate traces of water and stored at − 20 °C in a sealed brown bottle to avoid its photo-degradation until its use. The yield in essential oils was carried out in triplicate and expressed as a percentage by dry weight.

To determinate chemical composition of essential oil, chromatographic analyses were carried out using gas chromatography coupled to mass spectrometry (GC–MS) HP 7890 (II). EO identification was achieved by linking the recorded mass spectra with those saved in the Wiley 09 NIST 2011 NIST Mass Spectral Library of the GC–MS data system^23,24^.

### Clay mineral purification

The raw clay used in this study is collected from Zaghouan deposit (north-eastern Tunisia). A detailed study of the X-ray diffraction pattern (XRD) of this clay (Fig. 2) shows the presence of the characteristic reflections of calcite and quartz as impurities. Thus, its purification should be realized.

The raw montmorillonite (Mt) was first dried at 105 °C during 24 h to facilitate its grinding. The dried clay was crushed in a mortar with a pestle and then strained through a 60-mesh sieve. Zaghouan clay has been purified by sedimentation using the experimental protocol previously described in^25^. Finally, the montmorillonite fraction was treated with NaCl solution (1 M) for 24 h to generate the homoionic Na-montmorillonite (purified-clay).

### Characterization techniques

The chemical analysis of clay was performed by means of the MagiX X ray fluorescence (XRF) spectrometer of PANanytical**.**

X-ray powder diffraction (XRD) patterns have taken place on an automated X'Pert Pro MPD diffractometer with a primary monochromator Ge (111) (strictly monochromatic Cu Kα1 radiation) and a X'Celerator (Real Time Mul-tiple Strip) detector. The powder profiles were recorded between 2° and 70° in 2θ with a total measuring time of 30 min and at a scanning rate of 2°/min.

Bruker's Vertex70 FT-IR spectrophotometer allows the recording of FT-IR spectra of solid, liquid and gaseous samples in the near and mid-infrared range. In the present analysis, we worked by Attenuated Total Reflection (ATR). An accessory of the Golden Gate Single Reflection Diamond ATR System was used for this purpose. The samples were recorded without prior preparation. A standard spectral resolution of 4 cm^−1^ in the spectral range of 4000–500 cm^−1^ was used for spectrum acquisition, as well as 64 accumulations per sample.

The textural properties were evaluated from N_2_ adsorption–desorption at − 196 °C using an automatic ASAP 2420 system from Micromeritics. Firstly, samples were outgassed at 200 °C and 10^–4^ mbar overnight. Brunauer–Emmett–Teller (BET) equation was used to estimate the specific surface area (S_BET)_ by considering a N_2_ cross section of 16.2 Å. Micropore volume was calculated from the t-plot method from Lippens and De Boer calculations^26^. The total pore volume was determined from the adsorption isotherm at P/P_0_ = 0.996.

Elemental Analysis CHN was obtained with a LECO CHNS 932 analyser to determine the carbon content present in the hybrids through the combustion of the samples at 1100 °C in pure oxygen.

X-Ray Photoelectron Spectroscopy (XPS) was used to compare the surface of the hybrid and clay before the adsorption process with a Physical Electronic PHI 5700 spectrometer using non monochromatic Mg–Kα radiation (300 W, 15 kV and 1253.6 eV). The spectra obtained were registered with a constant pass energy values at 29.35 eV, by using a 720 µm diameter circular analysis area. The X-Ray Photoelectron Spectroscopy spectra obtained were processed with PHI ACESS ESCA-V6.0F software and analysed with *Multipak 8.2B* package. The binding energy values were referenced to C 1s signal (284.8 eV). Gauss-Lorentz curves and Shirley type background were submitted to determinate the binding energy.

The cation exchange capacity (CEC) of each sample was obtained by the Kjeldahl method^27^; CEC is expressed in milli-equivalents per gram of calcined sample. Briefly, 0.2 g of clay was suspended in 10 mL of ammonium acetate solution (1 M) and stirred for 12 h at 30 °C. Then, the solid was centrifuged at 2500 rpm for 10 min. In the next step, NH_4_^+^-clay was washed and centrifuged three times with the ammonium acetate solution and then three times with methanol. This step ensures the total displacement of exchangeable cations. After the last operation, the methanol is replaced by distilled water. Then, the sample is shaken for 2 h before carrying out the determination of nitrogen by the Kjedahl method, which consists in introducing the clay suspension in an apparatus of type UDK 142 after addition of 3 mL of NaOH at 30%. The sample is undergone to NH_3_-flow for 5–10 min, which bubbles in a solution of boric acid. Thus, the ammonia is titrated with a solution of boric acid (2% vol.) and 3 drops of mixed indicator (turning from red-violet to green). The titration of ammonia was carried out by back titration with a sulfuric acid solution (H_2_SO_4_). The cation exchange capacity of the samples is determined using this Eq. (1):

1 $${\text{CEC }} = \, \left( {{\text{V}}_{{\text{A}}} - {\text{V}}_{{\text{B}}} } \right)/{\text{m}}) \cdot {1}00$$

where V_A_ is the volume of H_2_SO_4_ that served the total nitrogen determination in the clay test, V_B_ is the volume of H_2_SO_4_ that served the determination of total nitrogen in the blank test while m is mass of clay in the prepared sample.

The reactions involved in the indirect analysis of the exchange capacity are:

$${\text{NH}}_{{4}}^{ + } + {\text{ OH}}^{ - } \to {\text{ NH}}_{{3}} \left( {\text{g}} \right) \, + {\text{ H}}_{{2}} {\text{O}}$$

$${\text{NH}}_{{3}} \left( {\text{g}} \right) \, + {\text{ H}}_{{3}} {\text{BO}}_{{3}} \to {\text{ NH}}_{{4}}^{ + } + {\text{H}}_{{2}} {\text{BO}}_{{3}}$$

$${\text{H}}_{{2}} {\text{BO}}_{{3}}^{ - } + {\text{ H}}^{ + } \to {\text{ H}}_{{3}} {\text{BO}}_{{3}}$$

Gas chromatography coupled to mass spectrometry (GC–MS) on a gas chromatograph HP 7890 (II) and HP 5975 mass spectrometer (Agilent Technologies, Palo Alto, CA, USA ) with an electron impact ionization of 70 eV, HP 5 MS column with dimensions of 30 m length; 0.25 mm diameter and 0.25 µm film thickness was operated at a programmed temperature of 40–280 °C at a rate of 5 °C/min. Helium was used as a carrier at a flow rate of 1.2 mL/min.

Quantitative analyses were obtained using a Hewlett–Packard 7890 chromatograph equipped with a flame ionization detector **(**FID**)** and an electronic pressure control injector. An HP Innowax capillary column (polyethylene glycol, 30 m × 0.25 mm, 0.25 μm film thickness) (Agilent) was used at carrier gas N_2_ with a flow rate of 1.6 mL/min and a split ratio of 1:60. The column temperature was programmed at 35 °C for 10 min, then heated to 205 °C at a rate of 2 °C/min, and finally kept constant at 205 °C for 10 min. Injector and detector temperatures were held at 250 and 300 °C, respectively.

### Preparation of purified clay/CEO hybrid

The preparation of purified clay/CEO hybrid was carried out via an adsorption procedure. Firstly, the adsorption isotherms of CEO on purified clay were studied at different times. Experimentally, an essential oil solution was prepared by diluting an amount of pure CEO in ethanol (99%) in order to obtain a concentration of 130 mg/mL. Then, 0.03 g of adsorbent amount was dispersed into 2 mL of essential oil solution and left in collared pill boxes for a specified duration of 0, 1, 3, 5, 7, 12, 18, 24 and 36 h, under permanent agitation at room temperature.

The influence of initial concentrations of essential oil solutions (C_0_) on the amount adsorbed by purified clay was studied using an equilibrium time of 5 h. After that, the solid phase was separated by centrifugation at 10,000 rpm during 10 min, and all the supernatants has been collected and analyzed by GC-FID to determine the two major compounds Carvone and Limonene concentration. The amounts of each two major compounds adsorbed per mass unit of adsorbent at equilibrium Q (mg/g) was calculated by following Eq. (2):

2 $$Q\left( {mg/g} \right) = \, V\left( { \, C_{0} - \, C_{e} } \right)/m_{ads}$$

where *C*_0*,*_* C*_*e*_ correspond to the initial concentration of adsorbate and the concentration of adsorbate at equilibrium (mg/L), respectively; *V* is the volume of adsorbate (L) and *m*_*ads*_ is the mass of the adsorbent (purified clay).

### Adsorption kinetics

Different models such as pseudo-first order and pseudo-second order have been applied to explain the kinetics of adsorption.

The rate constants for the adsorption of CEO onto purified clay were calculated using the two model expressions shown below.

The pseudo first order equation's linear form is given^28^ by Eq. (3), which was studied to describe the adsorption caused by ion exchange process.

3 $$\log \left( {q_{e} - q_{t} } \right) = \log q_{e} - \left( {\frac{{k_{1} }}{2.303}} \right)t$$

The pseudo second-order model's linear form is given^29^ by Eq. (4), which is centered on the assumption that the rate-limiting step is the number of active sites that could be occupied by the adsorbent.

4 $$\frac{t}{{q_{t} }} = \frac{1}{{k_{2} q_{e}^{2} }} + \frac{1}{{q_{e} }}t$$

where ${k}_{1}$ (1/min) is the equilibrium rate constant of pseudo first-order kinetics, ${k}_{2}$ (g/mg.h) is the equilibrium rate constant of adsorption of pseudo second-order kinetics, ${q}_{e}$ (mg/g) and ${q}_{t}$ (mg/g) are the amounts of Carvone and Limonene adsorbed at equilibrium and at a time t, respectively.

### Modeling of adsorption isotherms

To describe the adsorption equilibrium, Langmuir and Freundlich isotherm models were applied to the experimental data.

The equilibrium distribution of adsorbed ions between the clay and essential oil has been described by the Langmuir model^30^. This isotherm is applicable to monolayer adsorption on a surface with a defined number of similar sites. The linear equation of the Langmuir model is defined by Eq. (5):

5 $$\frac{{C_{e} }}{{Q_{ads} }} = \frac{1}{{Q_{M} \times K_{L} }} + \frac{{C_{e} }}{{Q_{M} }}$$

where Q_ads_ represents the amount of adsorbed Carvone or Limonene per unit mass of adsorbent (mg/g), ${C}_{e}$ is the equilibrium concentration of the solution (mg/mL), Q_M_ is the maximum adsorption capacity of Carvone or Limonene per unit mass of adsorbent to form a complete monolayer on the surface bound at high ${C}_{e}$, and ${K}_{L}$ is the corresponding Langmuir parameters.

Thus, curves giving $\frac{{{\varvec{C}}}_{{\varvec{e}}}}{{{\varvec{Q}}}_{{\varvec{a}}{\varvec{d}}{\varvec{s}}}}$ as a function of ${C}_{e}$ for purified clay allow the determination of Q_M_ and K_L_.

The adsorption on a heterogeneous surface has been described by the Freundlich model^31^ according to the formula (6) in its linear form:

6 $$LnQ_{ads } = LnK_{f } + \frac{1}{n} \times LnC_{e}$$

where n and ${K}_{f }\mathrm{}$ are Freundlich constants, where ${K}_{f}$ represents the adsorption capacity of clay and n gives an indication of the adsorption process favorability.

The linearization of the adsorption isotherms according to this model, is given in the curve $Ln{Q}_{ads}$ as a function of $Ln{C}_{e}$.

### Antibacterial activity

The antibacterial activity of CEO alone and purified clay/CEO hybrid^32^ was tested against Gram positive bacteria *Staphylococcus (S.) aureus* (ATCC 25,923) and maintained in the liquid growth LB medium at 30 °C. Moreover, the antifungal potential of this EO alone and purified clay/CEO hybrid was performed against *Candida albicans* (ATCC 12,321). Fungal culture was grown in yeast peptone dextrose (YPD) broth until reaching a cell density value of 105 cells per mL. Bacterial strain was obtained from the collection of the "Laboratory of Bioactive Substances, Biotechnology Center of Borj Cedria, Tunisia".

### Determination of the minimum inhibitory concentration (MIC)

The minimum inhibitory concentration is defined as the lowest concentration of sample sufficient to inhibit the growth of a strain in vitro.

The MIC is determined using cascade dilution method^23^ on liquid WB medium in eppendorfs tubes with a final volume of 500 µl.

Aliquots of the cell suspension of both *Staphylococcus (S.) aureus* (ATCC 25,923) and *Candida albicans (*ATCC 12,321) are added to 3.5 × 10^4^ (CFU / mL). With CFU, a Colony Forming Unit is used to estimate the number of strains.

Sample dilutions are made in ranges of the following concentrations:

- Purified clay concentrations range from 100 to 6.25 mg/mL.
- Concentrations of CEO and purified clay/CEO range from 910 to 14.21 mg/mL.

The test tubes are incubated at 30 °C for 24 h. The MIC values are determined as the lowest concentration of the sample that exhibits total inhibition of cell growth.

### Declaration statement

The confirm that all methods were carried out in accordance with relevant guidelines in the method section. The permit to collect plant materials for scientific research was accorded by the laboratory of bioactive substances, Biotechnology center of Borj Cedria, Tunisia.

## Results and discussion

### Caraway essential oil

**Figure 1:**
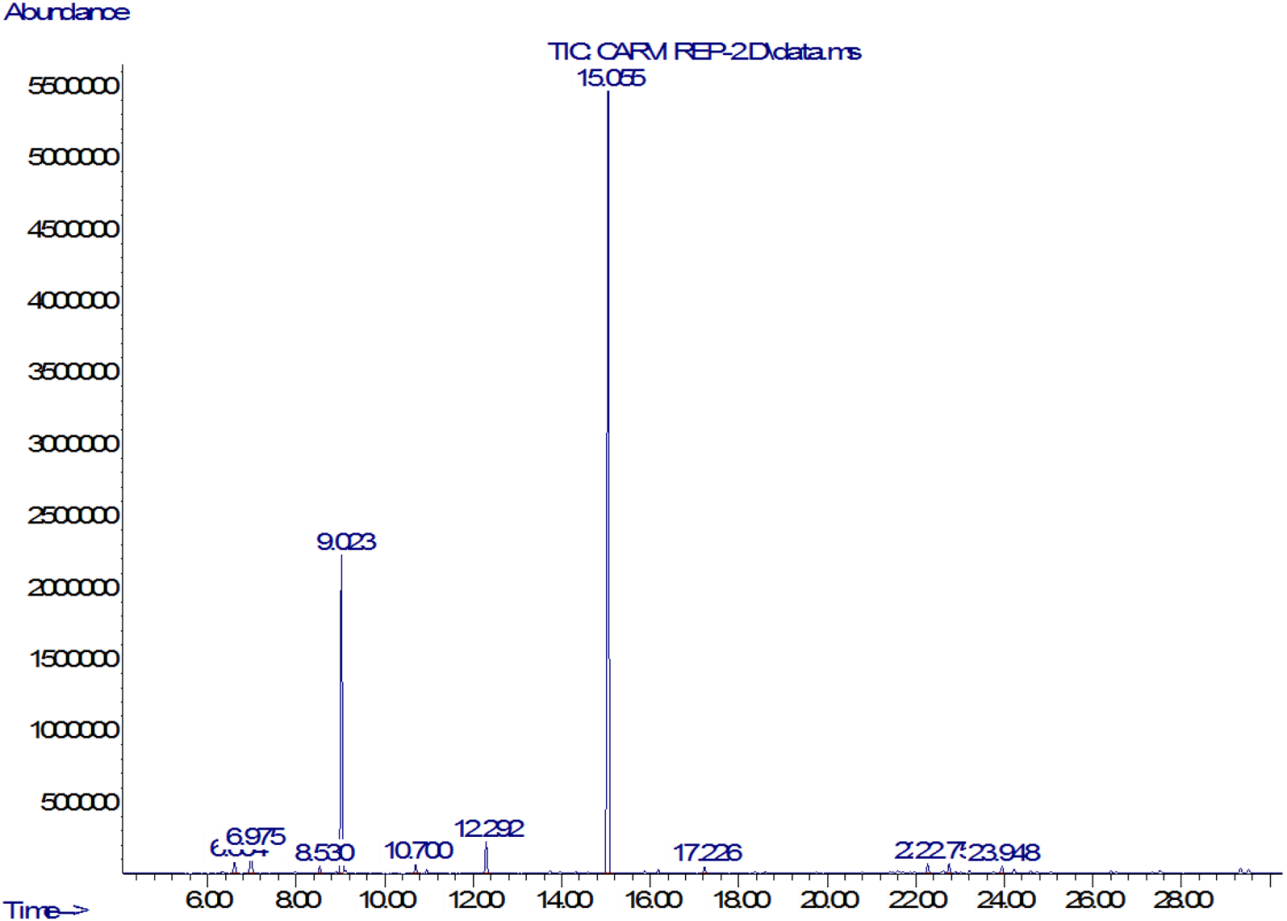
GC–MS spectra of CEO.

Figure 1 illustrates the Chromatographic spectrum of CEO characterized by an inhibitory concentration and a specific density equal to 125 mg/mL and 910 mg/mL, respectively. The spectrum shows that CEO composed of 11 constituents identified in Table 1. For adsorption studies, the two major constituents have been selected; Carvone (R-Carvone) was present in the highest percentage (68.30%) followed by Limonene (D-limonene) (22.54%), Trace amounts of other compounds including monoterpene hydrocarbons (Camphene (6.98%), $\alpha$-pinene (1.03%)), Oxygenated Monoterpenes (Camphor (2.38%)), Sesquiterpene Hydrocarbons (α-Selinene (0.95%), Delta-cadinene (0.85%)) Ester (Myrtenyl acetate (0.47%)), α -thujone (0.73%), Veridiflorol (0.56%), $\delta$.3-Carene (0.54%).

**Table 1:** Chemical composition of CEO by GC–MS.

Pic	Molecule	R_t_ (min)	Area (%)
1	$\alpha$-pinene	6.603	1.03
2	Camphene	2.04	6.975
3	Delta.3-Carene	8.531	0.54
4	D- limonene	9.022	22.54
5	$\alpha$–thujone	10.7	0.73
6	Camphor	12.290	2.38
7	R- Carvone	15.056	68.30
8	Myrtenyl acetate	17.225	0.47
9	$\delta$-cadinene	22.266	0.85
10	Selina-3,7(11)-diene	22.752	0.95
11	Veridiflorol	23.949	0.56

The results are compatible with previous reports obtained by^33^, who found that Chinese *Carum Carvi* essential oil contains (R)-Carvone (51.6%) and D-Limonene (38.26%) as its two main components. (R)-Carvone and D-limonene were also found to be the two main constituents in the EO collected from Europe which present more than 95% of the essential oil of caraway fruits and all of the other aroma compounds exist at trace levels only^34^. In addition, in North America, the amount attend 46.62% of Carvone and 45.49% Limonene^35^.

The slight variation in the percentages of Carvone and Limonene in the chemical composition of trace elements may be due to geographical and plant population variation.

In Addition, based to the comparative study of ^36^ between German, Egyptian and Tunisian CEO, it can confirm that the highest percentage of Carvone and Limonene (90.84%), observed in this study, indicates that CEO from Korba has a good quality. Thus, Carvone and Limonene are responsible for all CEO benefits^35^.

### Clay analyses

Clay analyses were performed to know the chemical compositions of the mineral phases present in the clay sample. XRF data, shown in Table 2, reveals that the solid is mainly an aluminosilicate^37^. The presence of Fe or Mg could be ascribed to the partial substitution of Al by these elements or the presence of some impurities^38^. The presence of Ca, Na or K could be ascribed to the presence of these cations in the interlayer space to counterbalance the charge deficiency of the sheets or by the existence of impurities^39^.

**Table 2:** Chemical composition (wt %) of purified clay.

	SiO_2_	Al_2_O_3_	Fe_2_O_3_	MgO	CaO	K_2_O	TiO_2_	Na_2_O	P_2_O_5_	BaO	ZnO	CuO
Wt%	53.72	24.41	8.41	4.08	2.49	1.58	1.14	0.815	0.27	0.1	0.04	0.02

**Figure 2:**
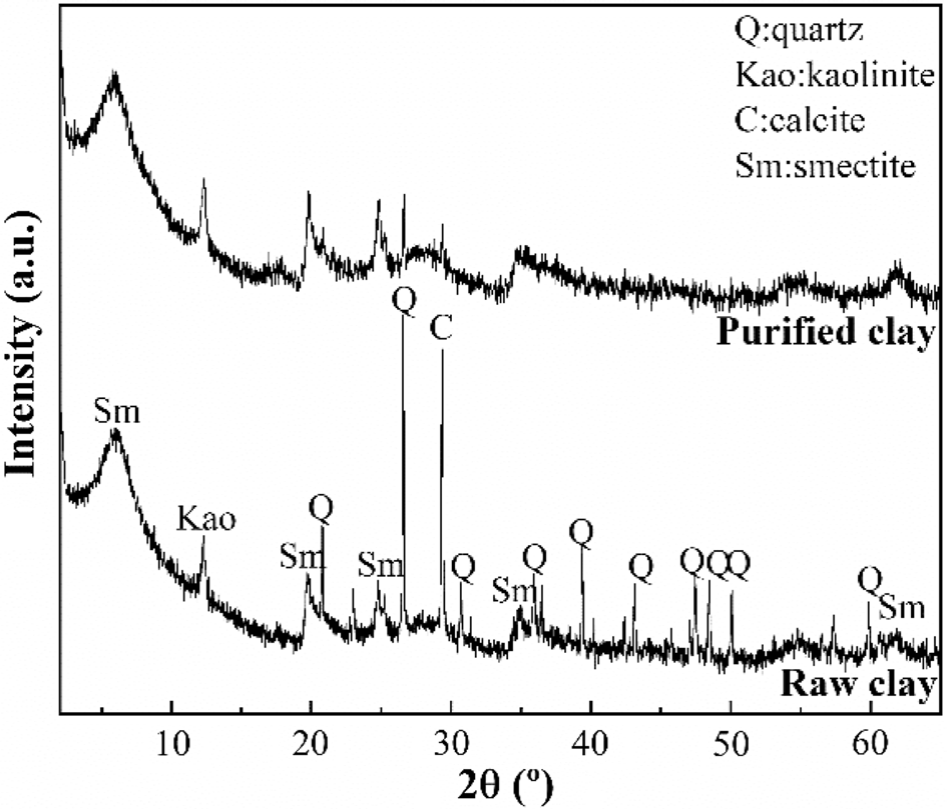
XRD patterns of the raw clay and purified clay.

Figure 2 presents XRD patterns of raw clay and purified clay. The X-ray diffractogram of raw clay shows a peak located at 2θ of 6.15°, which is assigned to the *d*_001_ reflection of montmorillonite. This implies a basal spacing of 14.3 Å, which is typical of smectites. It is not possible to discern between dioctahedral and trioctahedral smectites. For this purpose, it is possible to draw on the *d*_060_ reflection. The presence of a peak located at 62.3° 2θ (1.49 Å) confirms the presence of the typical dioctahedral sheet of montmorillonite. This result is in agreement with the data shown by XRF (Table 2). On the other hand, it is also noteworthy the presence of a signal located at 12.6° 2θ, which is attributed to the *d*_001_ reflection of kaolinite, another dioctahedral phyllosilicate whose basal spacing is of 7.14 Å^40^. Finally, it is also observed the presence of other minerals such as calcite and quartz in minor proportions, although both minerals are removed after the purification process by sedimentation.

**Figure 3:**
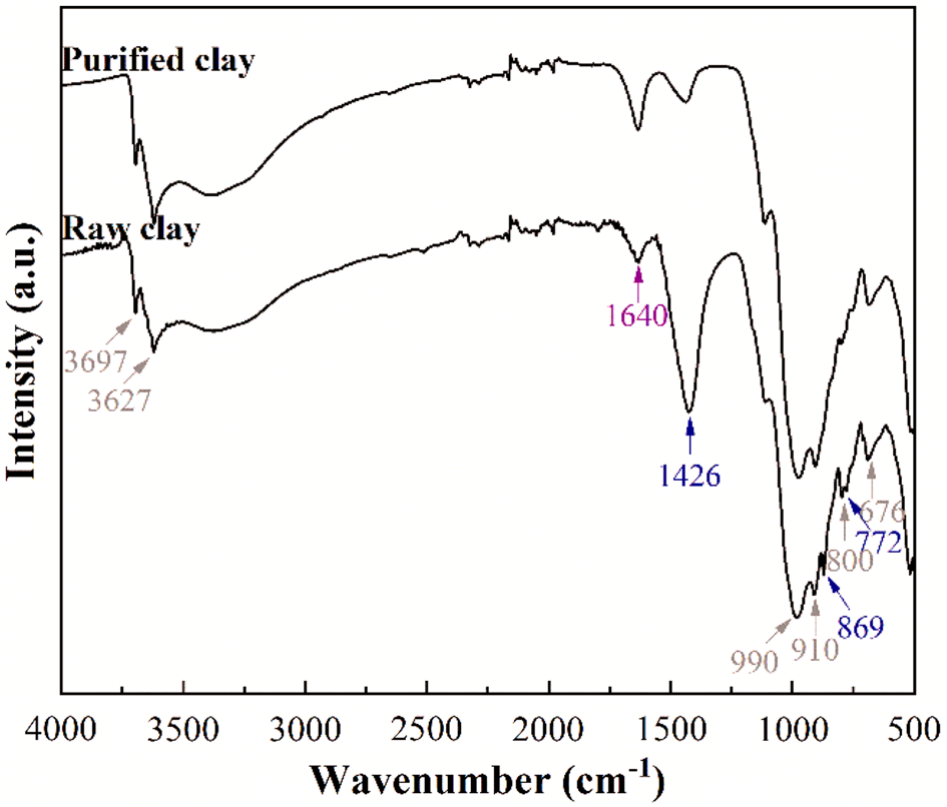
ATR spectra of ray clay and purified clay in the region of 4000–500 cm^−1^.

The ATR spectra of the raw clay and purified clay are shown in Fig. 3. In the OH–stretching region (3800–3000 cm^−1^), it can be observed two signals located at 3697 and 3627 cm^−1^^41^. The contribution with higher wavenumber is assigned to –OH stretching modes of Si–OH groups located on the external surface of the sheet. The band located at lower wavenumber is assigned to the –OH stretching modes of Al(OH)Si groups obtained due to the isomorphic substitution of Si^4+^ by Al^3+^-species^39^. Si–O stretching and bending modes as well as –OH bending adsorptions are compiled between 1300 and 500 cm^−1^. It can be observed how the main Si–O stretching vibrations show a maximum about 990 cm^−1^^42^. The band located about 910 cm^−1^ is assigned to typical Al_2_OH bending mode of dioctahedral smectites. On the other hand, it is also noteworthy the presence of other bands with high intensity, which are located at 1426, 869 and 772 cm^−1^, which are attributed to the presence of the asymmetric stretching, the out of-plane bending and the in plane bending modes of carbonate groups coming from calcite^43^ as was detected by XRD (Fig. 2). Finally, the band located about 1640 cm^−1^ is attributed to H–O–H bending band^44^.

**Figure 4:**
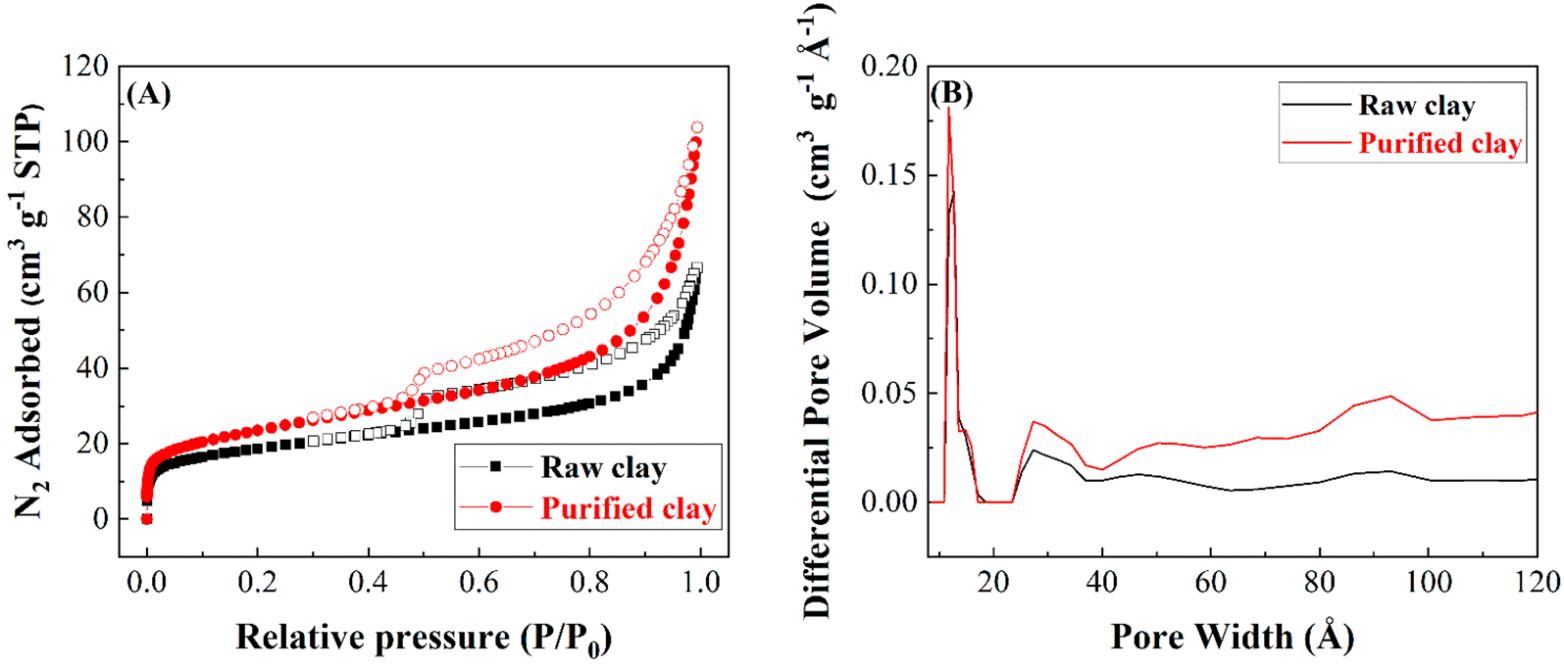
N_2_ adsorption–desorption isotherms at 196 °C of raw and purified clay (**A**). Pore size distribution of the raw and purified clay (**B**).

The textural properties of the raw clay and purified clay were determined from their N_2_ adsorption–desorption isotherms at − 196 °C (Fig. 4A). According to the IUPAC classification, both clays can be adjusted to type II^45^. The increase of the N_2_-adsorbed at high relative pressure is typical of microporous materials as a consequence of the N_2_-filling between adjacent clay particles. At the same way, it is also noteworthy the increase of the N_2_-observed at low relative pressure so these clays also must show microporosity. With regard to the hysteresis loop, both isotherms can be considered as H3, which is given by aggregates of plate-like particles as clays^45^.

The specific surface area (S_BET_) of raw and purified clay are shown in Table 3. These data reveal that the partial removal of calcite and quartz in the purification step causes a slight increase of the surface area. In any case, the S_BET_ values are in the same range of those achieved for other raw smectites such as montmorillonite or saponite in previous studies^46,47^. The analyses of the pore volume reveal that the purification treatment improves the micro- and mesoporosity.

**Table 3:** Textural parameters of raw clay and purified clay.

Samples	S_BET [m_^2^_g_^−1^_]_	t-plot_microp [m_^2^_g_^−1^_]_	V_p [cm_^3^_g_^−1^_]_	V_p(microp) [cm_^3^_g_^−1^_]_
Raw clay	66	33	0.06	0.016
Purified-clay	84	44	0.10	0.026

The pore size distribution was represented in Fig. 4B. The large anisotropic structure of the clay minerals is typical characteristic of these materials, leading to a broad pore size distribution. Thus, both clays show a relative narrow pore distribution with a maximum about 1.2 nm, which is attributed to the anisotropic structure of these materials and their respective voids between adjacent particles. As was shown in Table 3, the analysis of the pore size distribution favors the formation of micro and mesoporous probably due to the partial removal of calcite and quartz, which lack of porosity.

The cation exchange capacity of raw clay as well as the purified clay samples was measured by the ammonium acetate exchange According to the Kjeldhal method. The CEC is increased after purification from 61.11 to 76 meq/100 g of calcined clay; this increase can be attributed to the removal of the impurities present in the raw clay.

### Adsorption kinetics

**Figure 5:**
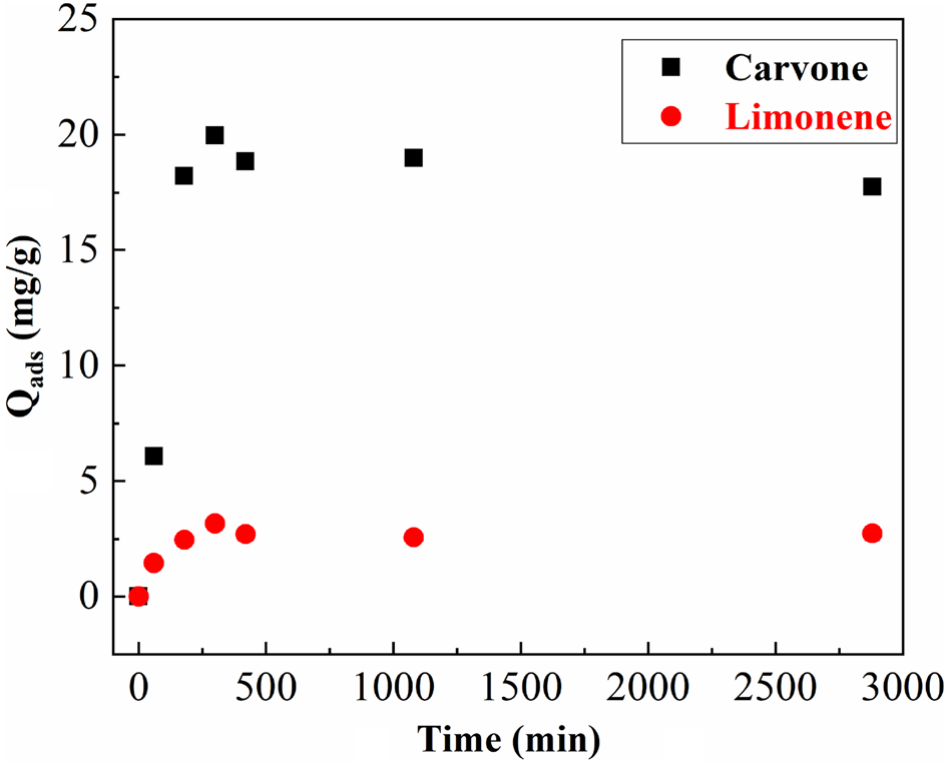
Effect of contact time Carvone and Limonene Adsorption onto purified clay using an initial CEO concentration 130 mg/mL at T = 25 °C.

The adsorption kinetic of two major compounds Carvone and Limonene on purified clay are shown in Fig. 5. The kinetic study display that a fast increase in the amount absorbed in the first hours for both compounds, reaching the equilibrium conditions after 300–360 min of treatment. In addition, Fig. 5 also shows how the amount adsorbed is higher for Carvone in comparison to Limonene. This result can be explicated by the abundance of Carvone in the crude essential oil (68.30%) compared to Limonene (22.54%). In addition the selectivity was improved according to the polarity of the terpenic component (Carvone) in comparison to the mono-terpene hydrocarbons (Limonene)^24^.

**Figure 6:**
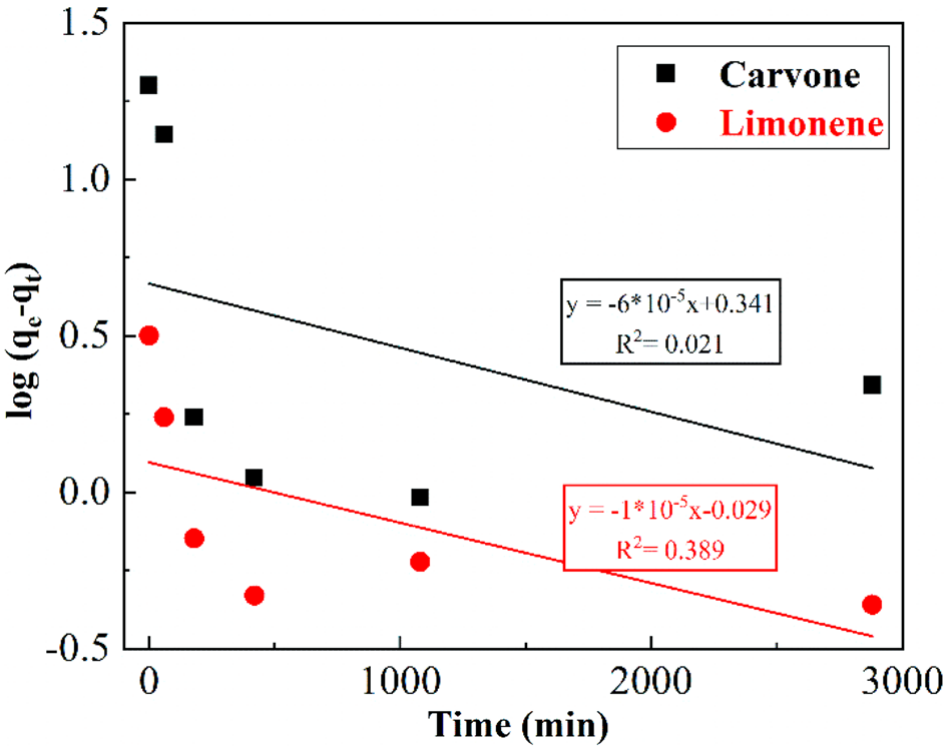
Pseudo-first-order kinetic model of Carvone and Limonene adsorption on purified montmorillonite.

**Figure 7:**
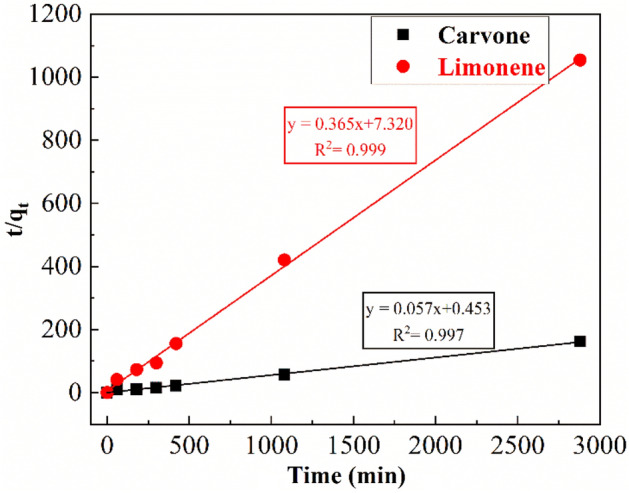
Pseudo-second-order kinetic model of Carvone and Limonene adsorption on purified montmorillonite.

The modeling of two major compounds adsorption kinetics on the purified montmorillonite was investigated using two common models (pseudo-first and pseudo-second-order models) (Figs. 6 and 7). Kinetic adsorption parameters for Carvone and Limonene with the corresponding correlation coefficients are presented in Table 4 where experimental equilibrium adsorption capacity q_e, exp_ can be determined experimentally from the curves (Fig. 5).

**Table 4:** Kinetic adsorption parameters according to the pseudo first and second-order models for Carvone and Limonene adsorption.

Compounds	q_e, exp_ (mg/g)	Pseudo-first-order kinetic model			Pseudo-second-order kinetic model		
		K_1_ (min^−1^)	q_e_ (mg/g)	R^2^	K_2_ (min^−1^)	q_e_ (mg/g)	R^2^
Carvone	19.98	0.14 10^−3^	2.2	0.0206	0.0068	18.0	0.9986
Limonene	3.05	0.23 10^−3^	0.9	0.3894	0.0187	2.7	0.9966

The correlation coefficient for the pseudo-second-order kinetic model was ${\mathrm{R}}_{\mathrm{Carvone}}^{2}$= 0.9986 and ${\mathrm{R}}_{\mathrm{Limonene}}^{2}$=0.9966 where the experimental q_e,exp_ values agreed with the calculated q_e_ values (Table 4) and, therefore, indicates that this model is the most suitable to the description of Carvone and Limonene adsorption by purified clay. Similar results was found in the adsorption of terpenic compounds from Eucalyptus globulus essential oil into beidellite, modified beidellite and organo-palygorskite^15,18^ his result suggests the adsorption process is controlled by the surface control through chemisorption^18^.

On the other hand, the comparative study of the equilibrium rate constant of pseudo second-order model ${\mathrm{k}}_{2}$ of Carvone and Limonene shows that limonene has the highest value (${\mathrm{k}}_{2\mathrm{ Limonene}}$ = 0.0187 min^−1^) however Carvone has the highest adsorbed amounts at equilibrium time (q_e_ = 18 mg/g) (Table 4).

### Concentration effect of the essential oil

**Figure 8:**
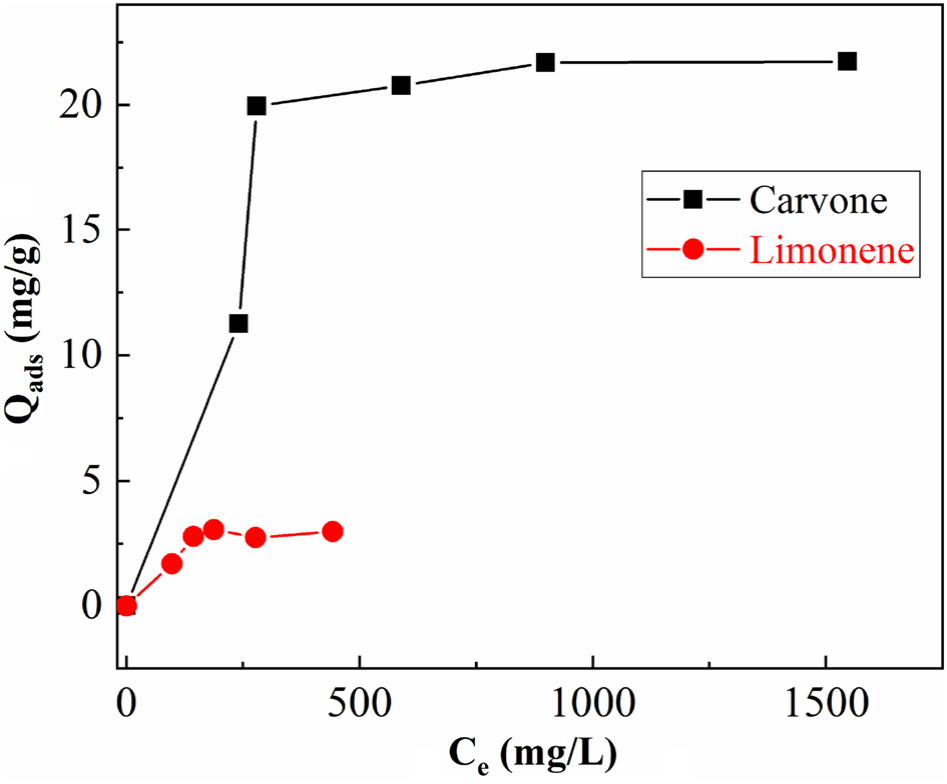
Adsorption isotherms of Carvone (**a**) and Limonene (**b**) onto purified clay (5 h and 0.03 g of purified clay).

The equilibrium adsorption of Carvone and Limonene on the purified montmorillonite at different initial concentration of CEO, under ideal time condition of 5 h of adsorption, and a mass of 0.03 g of clay (Fig. 8). The adsorption isotherms reveal that Carvone (19.98 mg/g) is preferably adsorbed than Limonene (3.05 mg/g). The higher adsorption must be ascribed to the presence of a ketone group in the Carvone, which can interact through hydrogen bonds with the silanol groups of the purified montmorillonite, as was proposed by previous adsorption models^16^. However, the absence of atoms with lone pair electrons in Limonene molecule limits its adsorption on the purified montmorillonite.

In order to carry out a more detailed study of the adsorption isotherms, both isotherms were fitted to Langmuir and Freundlich models (Table 5).

**Table 5:** Equilibrium adsorption parameters according to Langmuir and Freundlich models.

Compounds	Langmuir				Freundlich		
	Q_0_	Q_exp_	K_L_	R^2^	K_F_	1/n	R^2^
Carvone	24.21	19.98	0.007	0.9749	14.94	0.0003	0.3371
Limonene	3.3444	3.05	0.017	0.9477	2.04	0.001	0.3218

Based on the correlation coefficients R^2^_,_ it can be concluded that the Langmuir model is the adequate model to evaluate the adsorption capacity of Carvone and Limonene on montmorillonite (Table 5). Langmuir model assumes that the adsorption process takes place in the monolayer, where the adsorption of each molecule has equal activation energy with no interactions between adsorbed molecules. According to this model, we can note that the maximum of adsorption capacities Q_0_ was equal to 24.21 and 3.34 mg/g of Carvone and Limonene, respectively, are compatible with the experimental values. Carvone, the major element, showed the highest amount adsorbed although the K_L_ value, which defines the interaction adsorbate-adsorbent, is slightly higher for Limonene.

After the adsorption study, the obtained hybrid was characterized to confirm the adsorption of Carvone and Limonene.

### Evaluation of the bio-hybrid after the adsorption process

**Figure 9:**
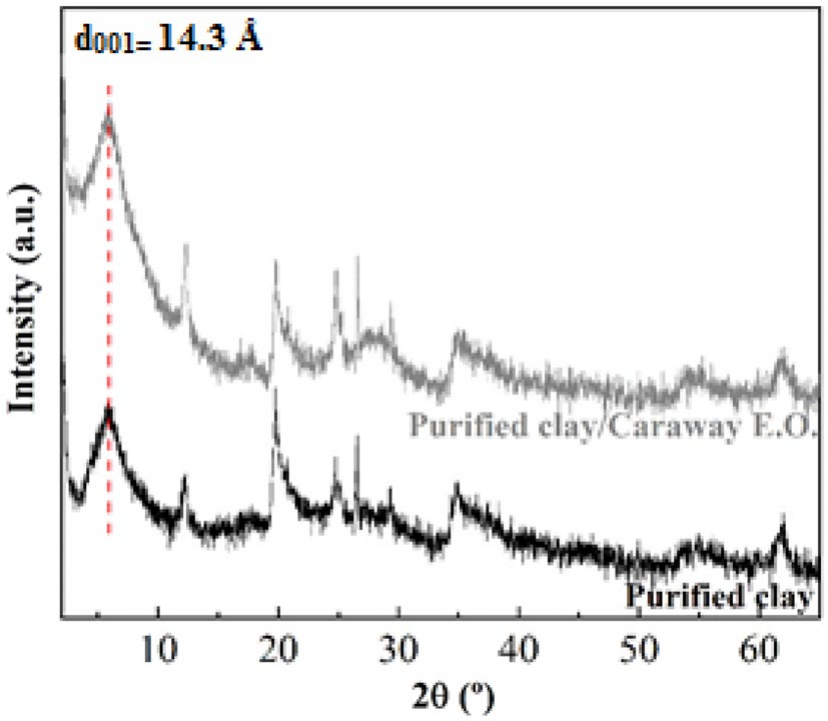
X-ray diffraction patterns for purified clay and purified clay/CEO.

Figure 9 presents the XRD patterns of the natural montmorillonite and hybrid. The X-ray diffractogram show that the hybrid pattern is similar to that reported for the purified montmorillonite before the adsorption in such a way that there are hardly any changes in the profiles of the diffractograms. The absence of changes in the d_001_ reflection suggests that Carvone and Limonene molecules are not intercalated in the interlayer space, which should cause an increase in the basal spacing of the purified montmorillonite. Thus, this results shows that both Carvone and Limonene must be adsorbed on the external surface of the purified montmorillonite^48^.

**Figure 10:**
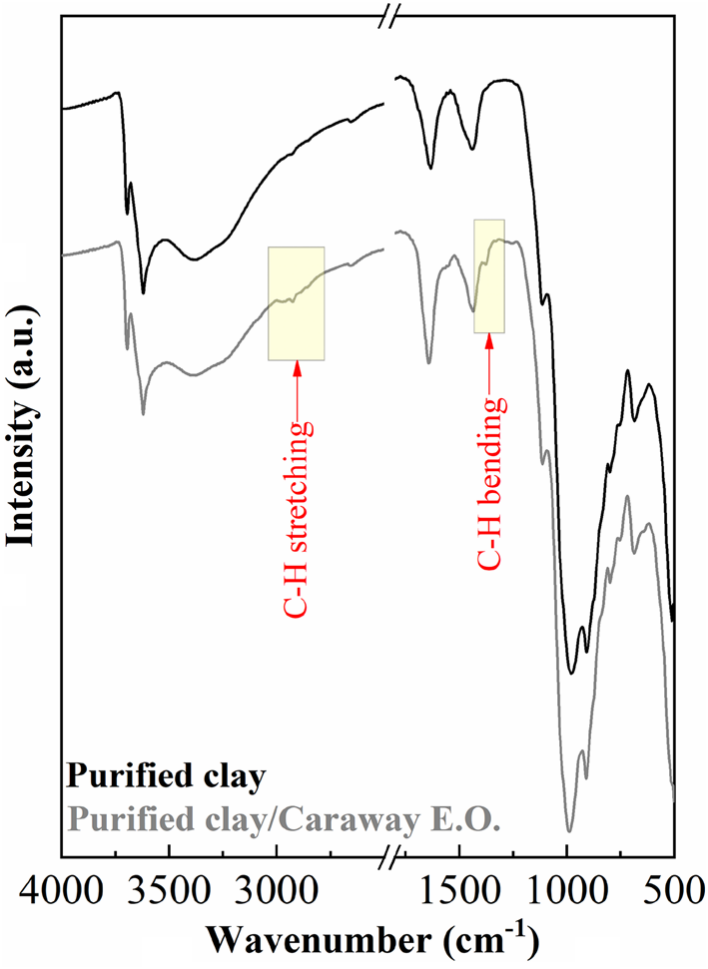
FT-IR spectra of purified clay and purified clay/CEO.

Figure 10 shows the FTIR spectrum of the purified montmorillonite after the adsorption process. This spectrum shows the arising of two small band located about 2925 and 2940 cm^−1^ , which are attributed to C–H stretching vibration modes coming from the adsorption of CEO in purified montmorillonite^19^.

In this sense, elemental analysis (CHN) was performed to corroborate the presence of Carvone and Limonene on the clay after the adsorption process Table 6. The CHN data reveals an increase in the amount of the carbon-content and the slightly increased amount of hydrogen on hybrid compared to purified montmorillonite, which are assigned to the successful adsorption of CEO into clay.

**Table 6:** Elemental analyses (CHN) of the starting materiel purified clay before and after adsorption of the CEO.

Sample	C (wt%)	H (wt%)	N (wt%)
Purified clay	0.3	0.7	0.03
Purified clay/CEO	2.1	0.9	0.03

In order to analyse the superficial composition of the purified montmorillonite and caraway hybrid, XPS measurements were carried out. Tables 7 and 8 compile the binding energies and atomic concentration of the adsorbents. The XPS data of the purified clay after the adsorption process show a slight increase of the atomic concentrations of C on the surface of the clay, confirming the adsorption of Carvone and Limonene on the surface of purified clay.

**Table 7:** Atomic concentrations of starting clay before and after the adsorption process of EO.

Samples	C 1s	Si 2p	O 1s	Al 2p	Fe 2p	Ca 2p	Mg 2p	Na 1s
Purified Clay	10.67	16.82	62.63	6.11	0.70	1.19	1.37	0.50
Hybrid	14.63	16.29	59.28	5.59	0.68	1.09	1.47	0.97

**Table 8:** Binding energies of starting clay before and after the adsorption process of EO.

Samples	C 1s	Si 2p	O 1s	Al 2p	Fe 2p	Ca 2p	Mg 2p	Na 1s
Purified Clay	284.8	102.6	531.8	74.5	712.3	348.4	49.7	1072.6
Hybrid	284.8	102.7	532.8	74.6	712.4	348.7	50.0	1072.6

### Anti-microbial activity

Results of minimum inhibitory concentration (MIC) test of purified clay, CEO and purified clay/CEO against *Staphylococcus aureus* strains and *C. albicans* was summarized in Table 9.

**Table 9:** Minimum inhibitory concentration tests of purified clay, CEO and Purified clay/CEO against Staphylococci aureus (S. aureus) and *Candida. Albicans.*

Samples	Antibacterial activity (mg/mL)	Anti-Candida activity (mg/mL)
Purified clay	NA	NA
CEO	125 ± 2.56	62.5 ± 1.42
Purified clay/CEO	62.5 ± 1.33	31.25 ± 2.56

NA, not actif.

The antibacterial activity of the CEO against *Staphylococcus aureus* strains showed a MIC value of 125 mg/mL. On the other side, purified clay did not register any activity. However, when encapsulated in clay, better antibacterial activity of hybrid was recorded (MIC = 62.5 mg/mL).

Furthermore, purified-clay/CEO hybrid showed a good activity against *C. albicans* yeast (MIC = 31.25 mg/mL). in this case, it is interesting to note that the anti-candidal activity of hybrid powder was 2 times greater than that registered in CEO alone (IC50 = 62.5 mg/mL).

From the obtained data, it can be observed how the addition of CEO to the inactive purified Clay, in optimised conditions, synergistically enhanced hybrid’s activity against *Staphylococcus aureus* and *Candida. albicans*. The stronger inhibitory effect can be explicated by the disruption of the plasma membrane of cells and the disorganisations of their mitochondrial structure^49^.

Due to the bacterial and Fungal-absorbed capability of purified clay, the bacteria were trapped in purified-clay/CEO composite which could make the complete contact between CEO and bacteria and fungi. Purified-clay/CEO combined the advantages of both purified clay and CEO in exerting the antibacterial and anti-fungal activity, and thus, the composite purified-clay/CEO has the high antibacterial and anti-fungal activity.

## Conclusions

The current study investigated proved that purified montmorillonite was successfully used as the carrier of Caraway essential oil in the preparation of the bio-hybrid purified clay/CEO for pharmaceutical uses.

The quantitative study by GC-FID of Carvone and Limonene reveals that a stirring for 5 h for a concentration of 130 mg/mL of EO and an amount of 0.03 g of clay leads to an optimal formation of the hybrid where the contents of Carvone and Limonene in purified clay/CEO are estimated to be about 19.98 mg/mL and 3.05 mg/g, respectively. Moreover, the kinetic study of the two major compounds adsorption on purified clay was best described by the pseudo-second-order model. Carvone and Limonene adsorption isotherms have been established to the Langmuir type. Furthermore, XRD, FT-IR, elemental analyses (CHN) and XPS characterization demonstrated the success adsorption of CEO in purified clay surface.

As a result of this work, the composite purified clay/CEO has a double antibacterial and antifungul activity compared to CEO. Thus, we have achieved the objectives of the research and obtained a biodegradeable, low cost and non-toxic hybrid powder which can be used in pharmaceutical industry.

## Data Availability

All data generated or analyzed during this study are included in this published article.
